# Comments on ‘Impact of Resistance Training and Chicken Intake on Vascular and Muscle Health in Elderly Women’ by Du et al. and Luo et al. – The Authors' Reply

**DOI:** 10.1002/jcsm.70153

**Published:** 2025-12-02

**Authors:** Shumpei Fujie, Naoki Horii, Hiroki Kajimoto, Henry Yamazaki, Kenichiro Inoue, Keiko Iemitsu, Masataka Uchida, Takuma Arimitsu, Yasushi Shinohara, Kiyoshi Sanada, Motohiko Miyachi, Motoyuki Iemitsu

**Affiliations:** ^1^ Faculty of Sport and Health Science Ritsumeikan University Shiga Japan; ^2^ Research Fellow of Japan Society for the Promotion of Science Tokyo Japan; ^3^ Faculty of Health Care, Undergraduate Department of Human Health Hachinohe Gakuin University Aomori Japan; ^4^ Faculty of Sport Sciences Waseda University Saitama Japan

We read with interest the comments by Du et al. [1] and Luo et al. [2] regarding our recent article ‘Impact of resistance training and chicken intake on vascular and muscle health in elderly women’ [3]. We are grateful for their thoughtful engagement with our study and their recognition of its contribution to understanding the effects of resistance training and high‐protein intake on human muscle and artery health status. Their relevant observations offer an excellent opportunity to further discuss the challenges of muscle and artery health status in human research.

In this study, to examine the effects of the combination of resistance training and high‐protein intake on the health status of aged muscles and arteries, we used a lot of materials and methods. Therefore, due to word count limitations, detailed descriptions of certain methods, such as ultrasound measurements of muscle thickness and echo intensity (EI), were provided in the Supplemental Materials and Methods. Separately, in this study, circulating creatinine levels were used as an index of renal function. No significant changes in circulating creatinine levels were observed before and after each intervention. Estimated glomerular filtration rate (eGFR) was analysed as an additional index of renal function (data not shown). The results showed no significant differences in eGFR before and after each intervention, and the percent changes in eGFR among the four groups were also not significant (Con: 5.0 ± 19.8%, HP: 6.1 ± 19.3%, RT: 1.6 ± 15.9%, HP + RT: −0.8 ± 24.2%), suggesting that these interventions did not affect renal function. Additionally, protein intake, as assessed by a questionnaire‐based dietary survey, did not change significantly before and after each intervention, indicating no excessive protein intake during the study. Therefore, these results suggest that the intervention in this study did not adversely affect renal function.

Our primary outcomes focused on physiological indices of artery health, and we assessed circulating angiotensin II (Ang II) as one of the potential underlying mechanisms. The results indicated that changes in Ang II might partially explain the effects of high‐protein diets on the increase in arterial stiffness induced by moderate to high‐intensity resistance training. However, the activity and expression of angiotensin‐converting enzyme (ACE) in arterial tissue were not assessed in this study, making it unclear whether Ang II alone could fully elucidate the underlying mechanism. Additionally, this study did not evaluate markers of oxidative stress and inflammation. Although circulating tumour necrosis factor‐α (TNF‐α) levels, a marker of inflammation, were measured, no significant changes before and after each intervention were observed among the four groups (data not shown in the manuscript). These findings suggest that the moderate to high‐intensity resistance training protocol employed in this study did not induce systemic inflammation. The underlying mechanisms, including oxidative stress and inflammation, were not the focus of this study and should be explored in future research.

In this study of our statistical analysis method, we opted for the Bonferroni test due to differences in sample sizes among the four groups caused by attrition. Additionally, we evaluated effect sizes by comparing the values of certain indices before and after intervention (data not shown in the manuscript) to assess the practical significance of the findings beyond mere statistical significance. According to Cohen [4], effect sizes are categorized as small (*d* = 0.2), medium (*d* = 0.5) and large (*d* ≥ 0.8). In this study, the primary outcomes were measures of vascular and muscle health, assessed by indices such as carotid β‐stiffness (index of arterial stiffness), carotid‐femoral pulse wave velocity (cfPWV; index of arterial stiffness), quadriceps cross‐sectional area (CSA; index of muscle mass) and one‐repetition maximum (1‐RM) strength (index of muscle strength). In this study, the effect size of carotid β‐stiffness in the resistance training (RT) group was 1.0, suggesting that moderate to high‐intensity resistance training increased arterial stiffness, whereas high‐protein intake mitigated this increase with a large effect size. In contrast, the effect size of cfPWV in the RT group was 0.2, indicating a small effect. Furthermore, the effect size of quadriceps CSA was 0.81 in the combination of resistance training and high‐protein intake (RT + HP) group, indicating a large effect. For 1‐RM, the effect sizes for leg extension were 1.55 in the RT group and 2.01 in the RT + HP group, whereas those for leg curl were 1.71 in the RT group and 1.69 in the RT + HP group. These findings suggest that the combination of high‐protein intake and moderate to high‐intensity resistance training enhances vascular and muscle health. Overall, most indices in this study demonstrated large effect sizes.

The primary finding of our study is that, for older adults, the combination of moderate to high‐intensity resistance training and regular intake of steamed chicken breast as a high‐protein food may be a novel exercise and nutritional therapy. Because the diagnostic criteria for sarcopenia primarily focus on lower limb muscle function and mass, improving the lower limbs represents the first strategy for reducing the risks of sarcopenia; therefore, in this study, we employed resistance training targeting the lower limbs. However, to ensure successful implementation in real‐world settings, it is crucial to emphasize the importance of designing personalized training programmes that incorporate both lower limb and upper body exercises to optimize individual outcomes. Once again, we are delighted that our research has garnered peer interest, and we sincerely thank Du et al. and Luo et al. for their valuable comments and kind attention to our work. We hope that this letter will lead to further discussions and fruitful exchanges in the future.

## Funding

This study was funded by the Ito Foundation, Tokyo, Japan (#134, M. Iemitsu). This work was supported by Grants‐in‐Aid for Scientific Research from the Ministry of Education, Culture, Sports, Science, and Technology of Japan (KAKENHI: 22H03487 for M. Iemitsu).

## Conflicts of Interest

The authors declare no conflicts of interest.
